# Cancer Moonshot 2020: a new march of clinical and translational medicine

**DOI:** 10.1186/s40169-016-0091-8

**Published:** 2016-03-10

**Authors:** Xiangdong Wang

**Affiliations:** Zhongshan Hospital Institute of Clinical Science, Fudan University, Shanghai, China; Shanghai Institute of Clinical Bioinformatics, Shanghai, China

The global scientific community is going to blast off to the Moon again with the clear aim to cure cancer 47 years after man first stepped onto the lunar surface. President Obama signed a Presidential Memorandum at the beginning of 2016—White House Cancer Moonshot Task Force—and called upon the world to “cure cancer once and for all”.

It is a new milestone and effort to re-unite scientific resources and experts, optimize therapeutic strategies and improve outcomes of patients with cancer. The Cancer Moonshot initiative delivers a number of opportunities and challenges, e.g., how can we integrate the Moonshot program with other anti-cancer strategies like clinical and translational medicine or precision medicine; how do we define focus points; how do we monitor progress along the “Moonshot” voyage?

The cancer research community is ready for the Moonshot 2020 program, although there are still a large number of thresholds, hurdles and obstacles to be determined and resolved along the way. A number of advanced biotechnologies in genomics, proteomics, metabolomics, systems biology, clinical bioinformatics and drug discovery have been translated into clinical practices and applications. Great potential lies in single-cell biology as well as in the study of the 3D architecture and organization of the genome, also known as gene repositioning. New therapies for gene editing, cell targeting, and immune approaches give the Cancer Moonshot 2020 program the ammunition required to reach its goals. For example, preclinical studies have shown the potential for multiplexed genome editing in in vitro and in vivo systems by CRISPR/Cas-mediated gene editing. Wang et al. simultaneously interrupted five distinct alleles in mouse embryonic stem cells, with an efficiency of 80 % to achieve the one-step generation of animals carrying mutations in multiple genes [1]. Transcription activator-like effector nucleases (TALENs) and clustered regularly interspaced short palindromic repeats (CRISPR)–Cas9 nucleases have been suggested as the most commonly employed approaches to edit the human genome.

Another exciting development is the application of antibodies in the inhibition of endogenous immune responses to cancer, known as checkpoint blockade therapy. Clinical trials have proved the safety and efficacy of antibodies that block the T cell inhibitory molecules CTLA-4 and PD-1 in treating subsets of patients with metastatic melanoma and renal cell carcinoma [2]. There is however quite some discussion about the types of cancer that would be most suitable for intervention using immunotherapy and there is great interest in specific biomarkers to monitor the various responses to these therapies.

We should also consider the potential for overlap and cooperation between the Cancer Moonshot 2020 program and clinical and translational medicine, and precision medicine. One part of the Cancer Moonshot 2020 program is to use progress in clinical and translational medicine, to re-unite, re-collect, re-organize, and re-optimize resources to fight diseases [3, 4].

Clinical and translational medicine was initiated 10 years ago to foster the communication between basic and clinical scientists, to translate advanced biotechnologies into clinical practice, and to improve the quality of life for patients. Precision medicine was announced in 2015 as a new emerging area and therapeutic strategy to improve the treatment and prognosis of patients by integrating clinical phenotypes with bioinformatics, computational science, mathematics, gene sequencing and systems biology.

Precision medicine was proposed, containing five critical elements: clinical bioinformatics, precision methodologies, disease-specific biomarkers, drug discovery and development, and precision regulations to guard the application of precision medicine [5]. A gene-based therapeutic strategy was initially suggested for inherited diseases and cancers on the basis of gene mutation and heterogeneity, and it was then later extended to include metabolic and cardiovascular diseases. Precision medicine could be one of the therapeutic strategies in the program of Cancer Moonshot 2020 program, or the program could be one of the therapeutic areas of focus in precision medicine.

The Cancer Moonshot initiative will promote the collaboration of all potential resources and it will improve the ability to:

- detect and prevent cancer at an early stage,
- develop cancer vaccination, immunotherapy, and combination therapy,
- perform genomic analysis of tumor and surrounding cells,
- allow enhanced data sharing,
- establish more oncology center of excellence, and
- develop more therapies for pediatric cancer [6].

The “White House Cancer Moonshot Task Force” was defined as a national program, government-led commission, Federal investments-dominated research, and cancer-focused therapy [7]. However, the fight against cancer is a global issue, which will greatly benefit from the involvement and contribution of the entire scientific community, focusing on a clear global strategy. To be able to effectively tackle the issues ahead, we need to develop a better understanding of cellular structures, organelles, functioning properties, metabolic circles, signaling pathways or interactions, and of the tumor microenvironment.

We should identify molecular targets during the discovery and development of target-based drugs and have biological function- and disease-specific biomarkers to monitor drug efficacy, efficiency and toxicology. We should have a better understanding of alterations of spatial genome organization and its effect on transcription as well as of higher-order chromosome folding and specific chromatin interactions in the topological mechanism of human cancer [8].

The Cancer Moonshot is a new ‘giant leap’ for clinical and translational medicine, a new opportunity to optimize scientific achievements and advances to cure cancer, with a special focus on cancer prevention and early detection and treatment.

The success of the Cancer Moonshot 2020 program will also be dependent upon the sharing of databases, which include clinical information, patient phenotypes, imaging, biochemical measurements, therapies, gene sequencing and systems biology.

We therefore believe that clinical and translational medicine will play an important role in the achievement of the goals set out in the Cancer Moonshot 2020 program. Cancer Moonshot “Apollo 11” has been launched and the anti-cancer spacecraft have started their voyage; the countdown to cure cancer has begun.
